# Decreased preoperative functional status is associated with increased mortality following coronary artery bypass graft surgery

**DOI:** 10.1371/journal.pone.0207883

**Published:** 2018-12-13

**Authors:** Hanjo Ko, Julius I. Ejiofor, Jessica E. Rydingsward, James D. Rawn, Jochen D. Muehlschlegel, Kenneth B. Christopher

**Affiliations:** 1 Department of Anesthesiology and Critical Care, University of Pennsylvania Health System, Philadelphia, Pennsylvania, United States of America; 2 Brigham and Women's Hospital, Division of Cardiac Surgery, Boston, Massachusetts, United States of America; 3 Brigham and Women's Hospital, Department of Rehabilitation, Boston, Massachusetts, United States of America; 4 Brigham and Women's Hospital, Department of Anesthesiology, Perioperative and Pain Medicine, Boston, Massachusetts, United States of America; 5 Brigham and Women's Hospital, The Nathan E. Hellman Memorial Laboratory, Renal Division, Channing Division of Network Medicine, Department of Medicine, Boston, Massachusetts, United States of America; Hospital Universitari Bellvitge, SPAIN

## Abstract

**Objectives:**

Functional status prior to coronary artery bypass graft surgery may be a risk factor for post-operative adverse events. We sought to examine the association between functional status in the 3 months prior to coronary artery bypass graft surgery and subsequent 180 day mortality.

**Design, setting, and participants:**

We performed a single center retrospective cohort study in 718 adults who received coronary artery bypass graft surgery from 2002 to 2014.

**Exposures:**

The exposure of interest was functional status determined within the 3 months preceding coronary artery bypass graft surgery. Functional status was measured and rated by a licensed physical therapist based on qualitative categories adapted from the Functional Independence Measure.

**Main outcomes and measures:**

The main outcome was 180-day all-cause mortality. A categorical risk prediction score was derived based on a logistic regression model of the function grades for each assessment.

**Results:**

In a logistic regression model adjusted for age, gender, New York Heart Association Class III/IV, chronic lung disease, hypertension, diabetes, cerebrovascular disease, and the Society of Thoracic Surgeons score, the lowest quartile of functional status was associated with an increased odds of 180-day mortality compared to patients with highest quartile of functional status [OR = 4.45 (95%CI 1.35, 14.69; P = 0.014)].

**Conclusions:**

Lower functional status prior to coronary artery bypass graft surgery is associated with increased 180-day all-cause mortality.

## Introduction

Risk assessment methods to predict postoperative mortality in cardiac surgical patients, such as the Society of Thoracic Surgeons (STS) score, are well established and are of great utility [1]. Limitations do exist in the ability to predict risk when age is utilized as a predictor of overall health [2]. Identification of high risk patients using a mix of self-reported and measured assessments of frailty is recently described [3, 4]. Though variably defined, a patient with frailty may have a mix of sarcopenia, cachexia, disability, decreased functional status, and numerous comorbidities [5].

While frailty closely correlates with ageing [6], it is an independent driver of adverse postoperative outcomes [7]. Frailty is strongly associated with major adverse cardiac and cerebrovascular events in cardiac surgery patients [3]. Addition of frailty or disability measures to cardiac surgery risk scores improves outcome discrimination in elderly patients [8]. Measured assessment of functional status by Physical Therapists utilizing the functional mobility sub scales adapted from the Functional Independence Measure (FIM) [9, 10] is a validated predictor of critical illness outcome [11].

While studies suggest that frailty is important for cardiac surgery outcomes, limited information exists on patient outcomes following coronary artery bypass graft surgery (CABG) in patients with low functional status prior to surgery. Exercise intervention or “prehab” prior to CABG is feasible [12, 13]. In low risk CABG patients, randomized implementation of a 10 week bi-weekly exercise program versus no intervention was shown to have small but significant decreases in hospital and Intensive Care Unit (ICU) length of stay in patients randomized to the exercise group [13]. We hypothesized that poor functional mobility status prior to CABG would be associated with increased mortality following cardiac surgery. To explore this hypothesis, we performed a single center observational cohort study of 718 adults from 2002 to 2014 who had a formal evaluation for functional mobility status by a physical therapist in the 90 days prior to CABG.

## Materials and methods

### Source population and data sources

We abstracted patient-level data from the Brigham and Women’s Hospital (BWH), a 793 bed teaching hospital in Boston, Massachusetts. Data on all CABG patients admitted to BWH between January 31, 2004 and December 22, 2014 were obtained through the Brigham Society of Thoracic Surgeons (STS) Adult Cardiac Surgery Database, Brigham Integrated Computing System [14] and the Research Patient Data Registry (RPDR) at Partners HealthCare [15]. Approval for the study was granted by the Partners Human Research Committee (Institutional Review Board). The Institutional Review Board waived the need for informed consent as data was analyzed anonymously.

### Study population

Patients were eligible for study inclusion if they were adults admitted to BWH as inpatients and received CABG during their hospitalization. During the study period, there were 6,082 individual patients, age ≥18 years, who underwent CABG. Exclusions included: 5,364 patients who did not receive a formal structured evaluation from a Physical Therapist within the 90 days prior to CABG. The physical function evaluation data is determined on inpatients following a physician order based on subjective assessment of frailty or concern over need for Physical Therapy services following hospital discharge. Thus, the analytic cohort was comprised of 718 patients.

### Exposure of interest and covariates

The exposure of interest was functional status prior to CABG defined as physical function assessed within the 90 days prior to CABG. Data was obtained from licensed physical therapists trained on the assessment of physical function based on qualitative categories adapted from the functional mobility sub scales of the Functional Independence Measure (FIM) [9, 10]. The traditional FIM data was not collected during assessment of physical function. The mobility sub scales incorporate transfers (including bed, chair, and wheelchair) as well as locomotion (including walking/wheelchair and stairs), and are scored on an ordinal scale based on percentage of active patient participation in the selected task [9]. The adapted scoring system grades patients on a scale of function with six designations from independent through dependent for motor tasks assessed, with a determination of not applicable used when a patient was either incapable of progressing to the designated task or for physical or medical limitations. The six designations were independent, standby assist/supervision, minimal assist, moderate assist, maximal assist, and total assist (**S1 Appendix**). Patients were assessed on bed mobility (roll side to side, supine to sit, sit to supine), transfers (sit to stand, stand to sit, bed to chair), and gait (level ambulation, stairs). The physical therapists were not aware of the study hypothesis, exposure or outcomes. From data physical therapy assesment data we have previously derived and validated an adapted FIM mobility sub scale score based clinical outcome prediction model in the hospital under study [11]. The cohort was stratified into quintiles according to the adapted mobility sub scale score [11] with the 2^nd^ and 3^rd^ quintile combined to achieve low, moderate and high functional status categories. The 2^nd^ and 3^rd^ quartile was combined based on the observation from our prior critical care study [11] that the largest differences in outcome are likely between the most functional versus the least functional patients. Covariates were chosen on the basis of published CABG short-term mortality models [1] and clinical experience. STS variable definitions are available at http://www.sts.org.

### End points

The primary end point was 180-day all-cause mortality following CABG. Secondary endpoints included 30 and 90-day post-CABG mortality.

### Assessment of mortality

Vital status was obtained from the Social Security Administration Death Master File which has high sensitivity and specificity for mortality [16]. We have validated the accuracy of the Social Security Administration Death Master File for in-hospital and out-of-hospital mortality in the RPDR database [17]. 100% of the cohort had at least 180-day follow up after hospital discharge. The censoring date was December 31, 2015.

### Power calculations

Previously, in a cohort of critically ill surgical patients (n = 6,304), we studied post-hospital mortality in ICU survivors who were assessed by physical therapists [11]. From these data, we assumed that 180-day post-discharge mortality would be 4-fold higher among the patients determined to have functional status in the lower 50% compared to those determined to have the top 50% of functional status. With an alpha error level of 5% and a power of 80%, the sample size required for our primary end point (180-day mortality) was 239 patients with the lower half of functional status and 239 patients with upper half of functional status.

### Descriptive statistics

Categorical variables were described by frequency distribution, and compared across outcome groups using contingency tables and chi-square testing. Continuous variables were examined graphically and in terms of summary statistics, and then compared across outcome groups using one-way analysis of variance or the Kruskal–Wallis test. Adjusted odds ratios were estimated by multivariable logistic regression models with inclusion of covariate terms thought to plausibly associate with both pre-existing functional status and 180-day post-CABG mortality. We individually tested for effect modification by functional status by adding an interaction term to the multivariate models.

The discriminatory ability of the clinical prediction model for 180-day mortality was quantified using the c-statistic. Calibration was assessed using the Hosmer-Lemeshow χ^2^ goodness-of-fit test and the accompanying p-value. The continuous adjusted relationship between preoperative functional status and risk of 180-day mortality post CABG was graphically represented utilizing the coefplot command [18]. In all analyses, p-values are two-tailed and values below 0.05 were considered statistically significant. All analyses were performed using STATA 14.1MP statistical software (StataCorp LP, College Station, TX). Data are reported in accordance with the guidelines outlined in Strengthening the Reporting of Observational Studies in Epidemiology [19].

## Results

Patient characteristics of the cohort were stratified according to 180-day mortality (**Table 1**). The mean age at hospital admission was 72.9 years. Most patients were male (64%), white (84%), with hypertension (78%) and a prior admission to the hospital under study within 90 days of CABG (97%). 35% of cohort patients had an NYHA class of III/IV. 98% underwent cardiopulmonary bypass with a mean (SD) bypass time of 133 minutes (83). 33% of the cohort underwent CABG and valvular surgery. 30, 90 and 180-day mortality rates were 2.6%, 3.6%, and 5.3%, respectively. Factors that were associated with 180-day mortality included valvular surgery, New York Heart Association Class III/IV, chronic lung disease, cerebrovascular disease, duration of cardiopulmonary bypass, and the STS Score (**Table 1**).

**Table 1:** Characteristics of the Cohort and Unadjusted Association of Potential Prognostic Determinants with 180-Day Mortality^a^.

	Alive N = 680	Expired^a^ N = 38	Total N = 718	P-value	Unadjusted OR (95%CI) for 180-day Mortality
*Age years-mean***±***SD*	**73.0 ± 9.5**	**72.2 ± 9.5**	**73.0 ± 9.5**	**0.61**^†^	**0.99 (0.96, 1.03)**
*Male Gender-no*.*(%)*	**435 (64)**	**28 (74)**	**463 (64)**	**0.22**	**1.58 (0.75, 3.30)**
*Non-White Race-no*.*(%)*	**106 (16)**	**4 (11)**	**110 (15)**	**0.40**	**0.64 (0.22, 1.83)**
*Valve Surgery-no*.*(%)*	**219 (32)**	**19 (50)**	**238 (33)**	**0.023**	**2.11 (1.09, 4.06)**
*New York Heart Association Class III/IV-no*.*(%)*	**226 (33)**	**27 (71)**	**253 (35)**	**<0.001**	**4.93 (2.40, 10.12)**
*Chronic Lung Disease-no*.*(%)*	**84 (12)**	**12 (32)**	**96 (13)**	**0.001**	**3.28 (1.59, 6.74)**
*Hypertension-no*.*(%)*	**530 (78)**	**32 (84)**	**562 (78)**	**0.36**	**1.51 (0.62, 3.68)**
*Number of Diseased Vessels-no*.*(%)*	**3.4 ± 0.8**	**3.3 ± 1.0**	**3.4 ± 0.8**	**0.31**^†^	**0.94 (0.64, 1.37)**
*Diabetes-no*.*(%)*	**222 (33)**	**13 (34)**	**235 (33)**	**0.84**	**1.07 (0.54, 2.14)**
*Chronic Kidney Disease-no*.*(%)*	**40 (6)**	**5 (13)**	**45 (6)**	**0.072**	**2.42 (0.90, 6.55)**
*Cerebro-Vascular Disease-no*.*(%)*	**79 (12)**	**12 (32)**	**91 (13)**	**<0.001**	**3.51 (1.70, 7.24)**
*Prior Cerebrovascular Accident-no*.*(%)*	**31 (5)**	**5 (13)**	**36 (5)**	**0.018**	**3.17 (1.16, 8.69)**
*Perfusion Time (min)-mean****±****SD*	**128.8 ± 67.2**	**203.4 ± 212.9**	**132.8 ± 83.3**	**<0.001**^†^	**1.01 (1.00, 1.01)**
*STS Score-median[IQR]*	**2 [1, 3]**	**4.5 [3, 8]**	**2 [1, 3]**	**<0.001**^‡^	**1.15 (1.08, 1.21)**

Data presented as n (%) unless otherwise indicated. P determined by chi-square except for ^†^ determined by ANOVA or ^‡^ determined by Kruskal-Wallis test.

a. Expired within 180-days of coronary artery bypass grafting

Stratification of the cohort into high, moderate and low functional status groups shows significant differences in age, STS Score and in-hospital mortality (**Table 2**).

**Table 2:** Patient characteristics by Functional Status group.

	Functional Status Group			
	High	Moderate	Low	P-value
*N*	**183**	**356**	**179**	
*Age years-mean***±***SD*	**73.3 ± 9.4**	**71.9 ± 9.9**	**74.6 ± 8.4**	**0.007**^†^
*Male Gender-no*.*(%)*	**115 (63)**	**236 (66)**	**112 (63)**	**0.60**
*Non-White Race-no*.*(%)*	**24 (13)**	**55 (15)**	**31 (17)**	**0.54**
*Valve Surgery-no*.*(%)*	**53 (29)**	**126 (35)**	**59 (33)**	**0.32**
*New York Heart Association Class III/IV-no*.*(%)*	**73 (40)**	**125 (35)**	**55 (31)**	**0.19**
*Chronic Lung Disease-no*.*(%)*	**26 (14)**	**47 (13)**	**23 (13)**	**0.92**
*Hypertension-no*.*(%)*	**136 (74)**	**282 (79)**	**144 (80)**	**0.31**
*Number of Diseased Vessels-no*.*(%)*	**3.5 ± 0.8**	**3.3 ± 0.8**	**3.4 ± 0.9**	**0.065**^**†**^
*Diabetes-no*.*(%)*	**59 (32)**	**117 (33)**	**59 (33)**	**0.99**
*Chronic Kidney Disease-no*.*(%)*	**14 (8)**	**22 (6)**	**9 (5)**	**0.59**
*Cerebro-Vascular Disease-no*.*(%)*	**24 (13)**	**41 (12)**	**26 (15)**	**0.60**
*Prior Cerebrovascular Accident-no*.*(%)*	**10 (5)**	**19 (5)**	**7 (4)**	**0.74**
*Perfusion Time (min)-mean****±****SD*	**129.1 ± 66.5**	**134.2 ± 90.1**	**134.0 ± 85.1**	**0.78**^**†**^
*STS Score-median[IQR]*	**2 [1, 3]**	**2 [1, 3]**	**3 [1, 4]**	**0.047**^‡^
*Prior CABG-no*.*(%)*	**6 (3.3)**	**11 (3.1)**	**9 (5.0)**	**0.51**
*Functional Status Score-mean****±****SD*^a^	**4.7 ± 4.7**	**18.7 ± 2.4**	**25.4 ± 1.8**	**<0.001**^†^
*In-hospital Mortality-no*.*(%)*	**2 (1)**	**7 (2)**	**10 (6)**	**0.015**
*180-day Mortality-no*.*(%)*^b^	**4 (2)**	**20 (6)**	**14 (8)**	**0.053**

Data presented as n (%) unless otherwise indicated. P determined by chi-square except for ^†^ determined by ANOVA or ^‡^ determined by Kruskal-Wallis test.

a. The Functional Status score is a severity of physical impairment risk score ranging from 0–29 points with 29 having the highest physical impairment

b. Expired within 180-days of coronary artery bypass grafting

Comparison of the 718 patient analytic cohort to the entire 6,082 patient parent cohort shows that small but significant differences exist with regards only to diabetes and the STS score. In-hospital and 180-day post-CABG mortality rates are not significantly different (**S2 Appendix**).

### Primary outcome

In the cohort, mortality in the 180 days after CABG was higher in patients with decreased functional status prior to CABG. The odds of 180-day mortality in patients with low functional status was 3.8 fold higher than patients with high functional status (**Table 3**).

**Table 3:** Unadjusted and adjusted associations between functional status category and 180-day mortality (N = 718).

	Functional Status Group		
	High	Moderate	Low
180-day mortality	OR (95% CI) P	OR (95% CI) P	OR (95% CI) P
*Crude*	1.00 (Referent)^a^	**2.66 (0.90, 7.91)** **0.078**	**3.80 (1.23, 11.77)** **0.021**
*Adjusted*^b^	1.00 (Referent)^a^	**3.31 (1.06, 10.35)** **0.040**	**4.45 (1.35, 14.69)** **0.014**
*Adjusted*^c^	1.00 (Referent)^a^	**3.20 (1.02, 10.02)** **0.046**	**4.36 (1.32, 14.39)** **0.016**
*Adjusted*^d^	1.00 (Referent)^a^	**3.33 (1.04, 10.65)** **0.043**	**4.42 (1.31, 14.85)** **0.016**

a. Referent in each case is the high functional status group

b. Model 1: Estimates adjusted for age, gender, New York Heart Association Class III/IV, Chronic Lung Disease, Hypertension, Diabetes, Cerebro-Vascular Disease, and the STS Score.

c. Model 2: Estimates adjusted for all covariates in Model 1 as well as Valve surgery.

d. Model 3: Estimates adjusted for all covariates in Model 1 as well as Perfusion Time.

**Fig 1:**
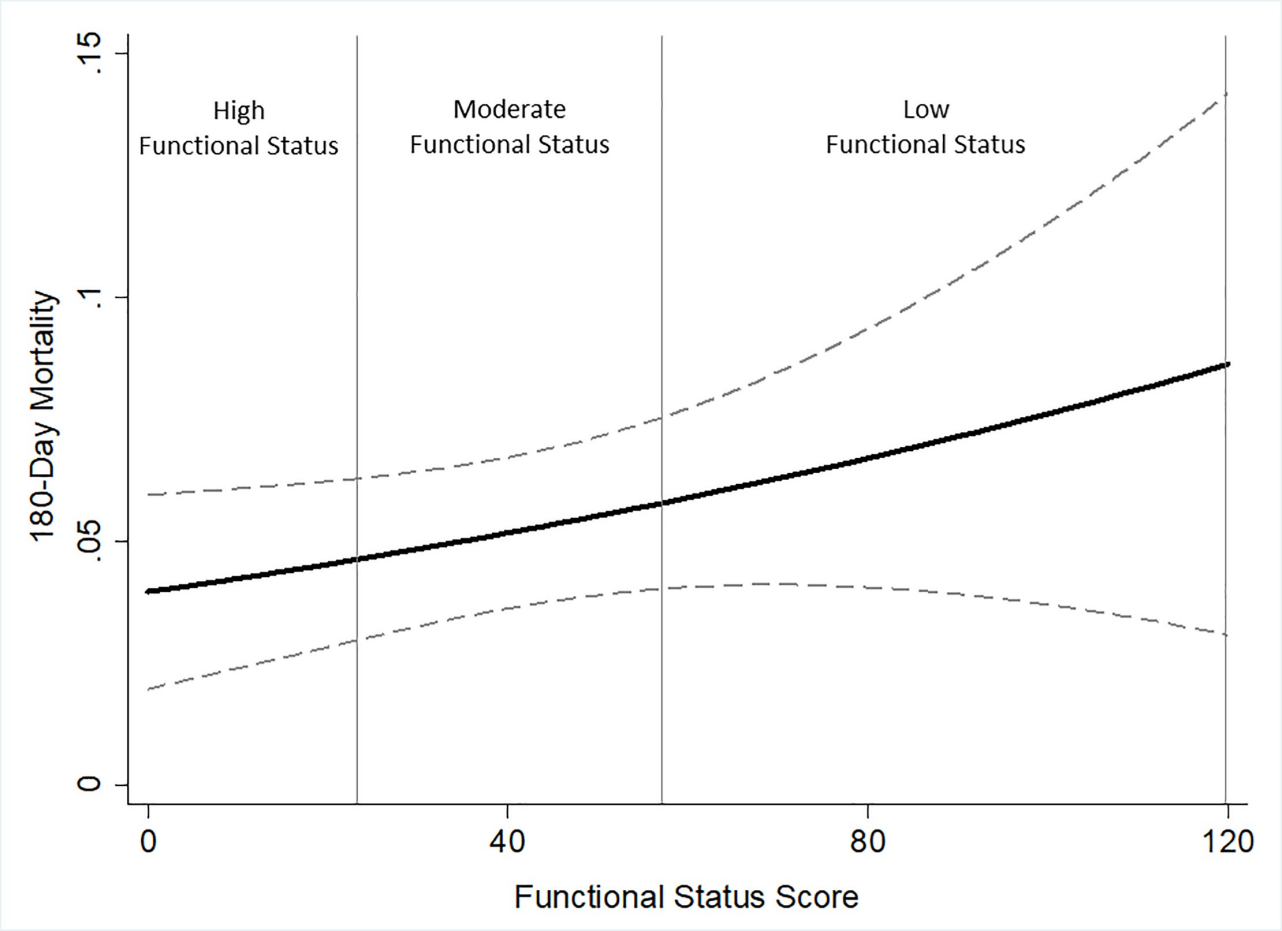
Adjusted association of functional status and 180-day mortality. Regression coefficient plot of multivariate estimates of the pre-CABG functional status-mortality association with 95% confidence intervals (dashes). Multivariate estimates adjusted for age, gender, New York Heart Association Class III/IV, Chronic Lung Disease, Hypertension, Diabetes, Cerebro-Vascular Disease and the STS Score.

Functional status remained a significant predictor of the odds of 180-day mortality after adjustment for age, gender, New York Heart Association Class III/IV, chronic lung disease, hypertension, diabetes, cerebrovascular disease and the STS Score. After adjustment, the odds of 180-day mortality in patients with moderate and low functional status was 3.3 and 4.5 fold higher respectively than patients with high functional status (**Table 3**). The adjusted 180-day mortality model showed good calibration (HL chi-squared 11.58, *P* = 0.17) and discrimination [c-statistic = 0.82 (95%CI 0.75–0.89)]. The coefficient plot of multivariate estimates demonstrates the increasing 180-day mortality with worsening of perioperative functional status (**Fig 1**).

Analysis of functional status in quintiles shows a similar relationship between pre-operative functional status and 180-day post-CABG mortality (**S3 Appendix)**. Further, assuming that CABG patients who were not assessed by a physical therapist had independent functional status, we analyzed the entire 6,082 patient parent cohort. We find that a similar relationship between low pre-operative functional status and elevated 180-day post-CABG mortality exists when unassessed CABG patients were considered the referent group (**S4 Appendix**).

In the cohort, there is no significant effect modification of the functional status-180-day mortality association on the basis of valvular surgery (P-interaction = 0.91), hospital length of stay (P-interaction = 0.99), evaluating physical therapist (P-interaction = 0.21), chronic kidney disease (P-interaction-0.80), year CABG was performed (P-interaction = 0.49) or chronic lung disease (P-interaction = 0.71). Effect modification is present regarding the duration of cardiopulmonary bypass time (P-interaction = 0.05). Adding the “duration of cardiopulmonary bypass” term to the final model does not alter the effect size or significance of the change in functional status-180 day mortality association (**Table 3 Model 3**).

## Discussion

In our cohort of adult CABG patients, we sought to characterize the relationship between functional status prior to CABG and subsequent 180-day mortality. Our data suggests that there is an increase risk of 180-day mortality in patients who undergo CABG with pre-existing decreased functional status. Our data supports that the performance of functional status evaluation prior to CABG can identify patients at high risk for subsequent adverse events. Patients with pre-ICU functional disability have heightened mortality in the year following ICU admission [20]. Hospitalization is associated with decline in functional status and independence [21]. Skeletal muscle atrophy can be demonstrated with more than 72 hours of immobilization in healthy subjects [22]. With prolonged bed rest, older adults show larger losses of muscle mass and strength relative to young adults [23]. In the critically ill, muscle mass loss and decreased strength are common complications [24].

In the critical care literature, low pre-ICU functional status is associated with increased mortality at one year [20]. Patient-perceived baseline functional status determined at ICU admission correlates with patient-perceived functional status of ICU survivors at 6 and 12 months after hospital discharge [25]. Frailty is a known driver of ICU survivorship and out of hospital outcomes [26]. Frailty closely correlates with ageing [6] and functional status changes due to critical illness likely differ by age [20]. Early physical therapy is shown to be safe in the critical care environment and can improve functional status [27]. Exercise training can improve functional capacity and measured muscle force in hospitalized patients [27]. Data from small trials indicate that preoperative physical therapy focused on respiratory muscle training may reduce post cardiac surgery atelectasis, pneumonia and length of stay [28]. As pre-existing functional status is a potentially modifiable risk factor, it is important to assess CABG candidates for functional status and frailty.

The potential limitations of this study are related to the observational design with inherent biases related to confounding as well as the lack of a randomly-distributed exposure. There is potential reverse causation, as the probability of 180-day mortality may be causally related to pre-CABG functional status where the frail may benefit most from CABG. Ascertainment bias is likely present as the study cohort had functional status measured as inpatients for reasons that may be absent in other CABG patients. As such, our study population may not be representative of the general CABG population. The study was performed in a single Boston tertiary care hospital and thus the results may not be generalizable to other acute care settings. Residual confounding may be present despite adjustment for multiple potential confounders. We are also unable to adjust for some variables that can alter functional status, including immobility and catabolism. Further, we do not have objective measures of sarcopenia [29].

The present study has several strengths and is unique in that it incorporates pre-existing functional status directly measured by a physical therapy practitioner to investigate 180-day mortality following CABG. Post-discharge mortality is validated in our research database (RPDR) under study [17]. In addition, we utilized validated assessments of cardiac surgery risk and have sufficient statistical power to detect clinically relevant differences in 180-day mortality.

In this single center study of 718 CABG patients, we conclude that decreased functional status prior to CABG is associated with increased mortality following CABG. Though our study cannot determine causation, our clinical data linking poor pre-existing functional status with worse clinical outcomes supports the rationale for physical therapy assessment before elective CABG. If our data is confirmed by others, the emphasis of strength maintenance or improvement prior to CABG should be part of a multidisciplinary effort to maximize the potential for recovery in adult CABG patients with moderate to low functional status.

## Supporting information

S1 Appendix Supplemental Table A. Scale of Function as determined by Physical Therapist.
(DOCX)

S2 Appendix Supplemental Table B. Characteristics of the Parent Cohort and the Analytic Cohort.
(DOCX)

S3 Appendix Supplemental Table C. Unadjusted and adjusted associations between functional status quintiles and 180-day mortality (N = 718).
(DOCX)

S4 Appendix Supplemental Table D. Unadjusted and adjusted associations between functional status groups and 180-day mortality in the parent cohort (N = 6,082).
(DOCX)

## References

[1] Shahian DM, O'Brien SM, Filardo G, Ferraris VA, Haan CK, Rich JB, et al The Society of Thoracic Surgeons 2008 cardiac surgery risk models: part 1—coronary artery bypass grafting surgery. Ann Thorac Surg. 2009;88(1 Suppl):S2–22. 10.1016/j.athoracsur.2009.05.053 . 19559822 10.1016/j.athoracsur.2009.05.053

[2] Green P, Woglom AE, Genereux P, Daneault B, Paradis JM, Schnell S, et al The impact of frailty status on survival after transcatheter aortic valve replacement in older adults with severe aortic stenosis: a single-center experience. JACC Cardiovasc Interv. 2012;5(9):974–81. 10.1016/j.jcin.2012.06.011 ; PubMed Central PMCID: PMCPMC3717525. 22995885 10.1016/j.jcin.2012.06.011 PMC3717525

[3] Sepehri A, Beggs T, Hassan A, Rigatto C, Shaw-Daigle C, Tangri N, et al The impact of frailty on outcomes after cardiac surgery: a systematic review. J Thorac Cardiovasc Surg. 2014;148(6):3110–7. 10.1016/j.jtcvs.2014.07.087 . 25199821 10.1016/j.jtcvs.2014.07.087

[4] Sundermann SH, Dademasch A, Seifert B, Rodriguez Cetina Biefer H, Emmert MY, Walther T, et al Frailty is a predictor of short- and mid-term mortality after elective cardiac surgery independently of age. Interact Cardiovasc Thorac Surg. 2014;18(5):580–5. 10.1093/icvts/ivu006 . 24497604 10.1093/icvts/ivu006

[5] Partridge JS, Harari D, Dhesi JK. Frailty in the older surgical patient: a review. Age and ageing. 2012;41(2):142–7. 10.1093/ageing/afr182 . 22345294 10.1093/ageing/afr182

[6] Fried LP, Tangen CM, Walston J, Newman AB, Hirsch C, Gottdiener J, et al Frailty in older adults: evidence for a phenotype. The journals of gerontology Series A, Biological sciences and medical sciences. 2001;56(3):M146–56. . 11253156 10.1093/gerona/56.3.m146

[7] Makary MA, Segev DL, Pronovost PJ, Syin D, Bandeen-Roche K, Patel P, et al Frailty as a predictor of surgical outcomes in older patients. J Am Coll Surg. 2010;210(6):901–8. 10.1016/j.jamcollsurg.2010.01.028 . 20510798 10.1016/j.jamcollsurg.2010.01.028

[8] Afilalo J, Mottillo S, Eisenberg MJ, Alexander KP, Noiseux N, Perrault LP, et al Addition of frailty and disability to cardiac surgery risk scores identifies elderly patients at high risk of mortality or major morbidity. Circulation Cardiovascular quality and outcomes. 2012;5(2):222–8. 10.1161/CIRCOUTCOMES.111.963157 . 22396586 10.1161/CIRCOUTCOMES.111.963157

[9] Granger CV, Hamilton BB, Zielezny M, Sherwin FS. Advances in functional assessment in medical rehabilitation. Topics in Geriatric Rehabilitation. 1986;1(3):59–74.

[10] Dodds TA, Martin DP, Stolov WC, Deyo RA. A validation of the functional independence measurement and its performance among rehabilitation inpatients. Archives of physical medicine and rehabilitation. 1993;74(5):531–6. . 8489365 10.1016/0003-9993(93)90119-u

[11] Rydingsward JE, Horkan CM, Mogensen KM, Quraishi SA, Amrein K, Christopher KB. Functional Status in ICU Survivors and Out of Hospital Outcomes: A Cohort Study. Crit Care Med. 2016;44(5):869–79. 10.1097/CCM.0000000000001627 ; PubMed Central PMCID: PMCPMC4833588. 26929191 10.1097/CCM.0000000000001627 PMC4833588

[12] Sawatzky JA, Kehler DS, Ready AE, Lerner N, Boreskie S, Lamont D, et al Prehabilitation program for elective coronary artery bypass graft surgery patients: a pilot randomized controlled study. Clin Rehabil. 2014;28(7):648–57. 10.1177/0269215513516475 . 24459173 10.1177/0269215513516475

[13] Arthur HM, Daniels C, McKelvie R, Hirsh J, Rush B. Effect of a preoperative intervention on preoperative and postoperative outcomes in low-risk patients awaiting elective coronary artery bypass graft surgery. A randomized, controlled trial. Ann Intern Med. 2000;133(4):253–62. . 10929164 10.7326/0003-4819-133-4-200008150-00007

[14] Teich JM, Glaser JP, Beckley RF, Aranow M, Bates DW, Kuperman GJ, et al The Brigham integrated computing system (BICS): advanced clinical systems in an academic hospital environment. International journal of medical informatics. 1999;54(3):197–208. . 10405879 10.1016/s1386-5056(99)00007-6

[15] Murphy SN, Chueh HC. A security architecture for query tools used to access large biomedical databases. Proc AMIA Symp. 2002:552–6. Epub 2002/12/05. D020002391 [pii]. ; PubMed Central PMCID: PMC2244204. 12463885 PMC2244204

[16] Sohn MW, Arnold N, Maynard C, Hynes DM. Accuracy and completeness of mortality data in the Department of Veterans Affairs. Popul Health Metr. 2006;4:2 Epub 2006/04/12. 1478-7954-4-2 [pii] 10.1186/1478-7954-4-2 ; PubMed Central PMCID: PMC1458356. 16606453 10.1186/1478-7954-4-2 PMC1458356

[17] Zager S, Mendu ML, Chang D, Bazick HS, Braun AB, Gibbons FK, et al Neighborhood poverty rate and mortality in patients receiving critical care in the academic medical center setting. Chest. 2011;139(6):1368–79. Epub 2011/04/02. chest.10-2594 [pii] 10.1378/chest.10-2594 ; PubMed Central PMCID: PMC3109648. 21454401 10.1378/chest.10-2594 PMC3109648

[18] Jann B. Plotting regression coefficients and other estimates. The Stata Journal. 2014;14(4):708–37.

[19] Vandenbroucke JP, von Elm E, Altman DG, Gotzsche PC, Mulrow CD, Pocock SJ, et al Strengthening the Reporting of Observational Studies in Epidemiology (STROBE): explanation and elaboration. Ann Intern Med. 2007;147(8):W163–94. 10.7326/0003-4819-147-8-200710160-00010-w1 . 17938389 10.7326/0003-4819-147-8-200710160-00010-w1

[20] Ferrante LE, Pisani MA, Murphy TE, Gahbauer EA, Leo-Summers LS, Gill TM. Functional Trajectories Among Older Persons Before and After Critical Illness. JAMA Intern Med. 2015 10.1001/jamainternmed.2014.7889 . 25665067 10.1001/jamainternmed.2014.7889 PMC4467795

[21] Sager MA, Franke T, Inouye SK, Landefeld CS, Morgan TM, Rudberg MA, et al Functional outcomes of acute medical illness and hospitalization in older persons. Arch Intern Med. 1996;156(6):645–52. . 8629876

[22] Kortebein P, Ferrando A, Lombeida J, Wolfe R, Evans WJ. Effect of 10 days of bed rest on skeletal muscle in healthy older adults. JAMA. 2007;297(16):1772–4. 10.1001/jama.297.16.1772-b . 17456818 10.1001/jama.297.16.1772-b

[23] Iannuzzi-Sucich M, Prestwood KM, Kenny AM. Prevalence of sarcopenia and predictors of skeletal muscle mass in healthy, older men and women. The journals of gerontology Series A, Biological sciences and medical sciences. 2002;57(12):M772–7. . 12456735 10.1093/gerona/57.12.m772

[24] Puthucheary ZA, Rawal J, McPhail M, Connolly B, Ratnayake G, Chan P, et al Acute skeletal muscle wasting in critical illness. JAMA. 2013;310(15):1591–600. 10.1001/jama.2013.278481 . 24108501 10.1001/jama.2013.278481

[25] Rodriguez-Villar S, Fernandez-Mendez R, Adams G, Rodriguez-Garcia JL, Arevalo-Serrano J, Sanchez-Casado M, et al Basal functional status predicts functional recovery in critically ill patients with multiple-organ failure. Journal of critical care. 2015;30(3):511–7. 10.1016/j.jcrc.2015.02.006 . 25817326 10.1016/j.jcrc.2015.02.006

[26] Bagshaw SM, Stelfox HT, McDermid RC, Rolfson DB, Tsuyuki RT, Baig N, et al Association between frailty and short- and long-term outcomes among critically ill patients: a multicentre prospective cohort study. CMAJ: Canadian Medical Association journal = journal de l'Association medicale canadienne. 2014;186(2):E95–102. 10.1503/cmaj.130639 ; PubMed Central PMCID: PMC3903764. 24277703 10.1503/cmaj.130639 PMC3903764

[27] Burtin C, Clerckx B, Robbeets C, Ferdinande P, Langer D, Troosters T, et al Early exercise in critically ill patients enhances short-term functional recovery. Crit Care Med. 2009;37(9):2499–505. 10.1097/CCM.0b013e3181a38937 . 19623052 10.1097/CCM.0b013e3181a38937

[28] Hulzebos EH, Smit Y, Helders PP, van Meeteren NL. Preoperative physical therapy for elective cardiac surgery patients. Cochrane database of systematic reviews. 2012;11:CD010118 10.1002/14651858.CD010118.pub2 . 23152283 10.1002/14651858.CD010118.pub2 PMC8101691

[29] Evans W. Functional and metabolic consequences of sarcopenia. J Nutr. 1997;127(5 Suppl):998S–1003S. 10.1093/jn/127.5.998S . 9164283 10.1093/jn/127.5.998S

