# Supplementary material for: Decreased preoperative functional status is associated with increased mortality following coronary artery bypass graft surgery
Source: PLoS One. 2018 Dec 13;13(12):e0207883. doi: 10.1371/journal.pone.0207883 (PMC6292581; doi:10.1371/journal.pone.0207883)
Supplement: S1 Appendix — Scale of Function as determined by Physical Therapist. (DOCX) [file pone.0207883.s001.docx]

| **Scale of Function** | **Definition** |
| --- | --- |
| Independent | No physical or cognitive assist although patients were allowed to utilize appropriate assistive devices as needed |
| Stand by | Required no physical assist, but demonstrates either the potential for motor/balance deficits or required verbal cueing for safety |
| Minimum | Patient performs 75% or greater of the activity |
| Moderate | Patient performs 50% or greater of the activity |
| Maximum | Patient performs 25% or greater of the activity |
| Total | Patient performs less than 25% of the activity or requires the assist of 2 skilled practitioners for safety |
| Not applicable | Patient was either incapable of progressing to the designated task or had physical or medical limitations preventing evaluation |

**S1 Appendix**

**Supplemental Table A: Scale of Function as determined by Physical Therapist**
