# Supplementary material for: Decreased preoperative functional status is associated with increased mortality following coronary artery bypass graft surgery
Source: PLoS One. 2018 Dec 13;13(12):e0207883. doi: 10.1371/journal.pone.0207883 (PMC6292581; doi:10.1371/journal.pone.0207883)
Supplement: S2 Appendix — Characteristics of the Parent Cohort and the Analytic Cohort. (DOCX) [file pone.0207883.s002.docx]

**S2 Appendix**

**Supplemental Table B: Characteristics of the Parent Cohort and the Analytic Cohort**

|  | **No Physical Therapist evaluation**  **N=5,364** | **Analytic Cohort**  **N=718** | **Parent Cohort**  **N=6,082** | **P-value** |
| --- | --- | --- | --- | --- |
| *Age years-mean***±***SD* | 69.0 ± 10.8 | 69.2 ± 10.8 | 69.1 ± 10.8 | 0.75^†^ |
| *Male Gender-no.(%)* | 3,806 (71) | 528 (74) | 4,334 (71) | 0.15 |
| *Non-White Race-no.(%)* | 868 (16) | 101 (14) | 969 (16) | 0.15 |
| *Valve Surgery-no.(%)* | 1,862 (35) | 238 (33) | 2,100 (35) | 0.41 |
| *New York Heart Association Class III/IV-no.(%)* | 2,017 (38) | 253 (35) | 2,270 (37) | 0.22 |
| *Chronic Lung Disease-no.(%)* | 707 (13) | 96 (13) | 803 (13) | 0.89 |
| *Hypertension-no.(%)* | 4,256 (79) | 562 (78) | 4,818 (79) | 0.51 |
| *Number of Diseased Vessels-no.(%)* | 3.4 ± 0.8 | 3.4 ± 0.8 | 3.4 ± 0.8 | 0.28^†^ |
| *Diabetes-no.(%)* | 1,963 (37) | 235 (33) | 2,198 (36) | 0.043 |
| *Cerebro-Vascular Disease-no.(%)* | 772 (14) | 91 (13) | 863 (14) | 0.22 |
| *Prior Cerebrovascular Accident-no.(%)* | 338 (6) | 36 (5) | 374 (6) | 0.18 |
| *Perfusion Time (min)-mean****±****SD* | 133.8 ± 70.6 | 132.9 ± 83.2 | 133.7 ± 72.3 | 0.78^†^ |
| *STS Score-median[IQR]* | 2 [1, 4] | 2 [1, 4] | 2 [1, 4] | 0.035^‡^ |
| *STS Score-mean****±****SD* | 3.5 ± 5.0 | 3.0 ± 4.0 | 3.4 ± 4.9 | 0.035^†^ |
| *In-hospital Mortality-no.(%)* | 176 (3) | 18 (3) | 194 (3) | 0.27 |
| *30-day Mortality-no.(%)* | 166 (3) | 19 (3) | 185 (3) | 0.51 |
| *180-day Mortality-no.(%)* | 309 (6) | 39 (5) | 348 (6) | 0.72 |

Data presented as n (%) unless otherwise indicated. P determined by chi-square except for ^†^ determined by

ANOVA or ^‡^ determined by Kruskal-Wallis test.
