# Supplementary material for: Decreased preoperative functional status is associated with increased mortality following coronary artery bypass graft surgery
Source: PLoS One. 2018 Dec 13;13(12):e0207883. doi: 10.1371/journal.pone.0207883 (PMC6292581; doi:10.1371/journal.pone.0207883)
Supplement: S3 Appendix — Unadjusted and adjusted associations between functional status quintiles and 180-day mortality (N = 718). (DOCX) [file pone.0207883.s003.docx]

**S3 Appendix**

**Supplementary Table C. Unadjusted and adjusted associations between functional**

**status quintiles and 180-day mortality (N= 718)**

**Functional Status Group**  .

|  | **High** | **Moderate-High** | **Moderate-Low** | **Low** |
| --- | --- | --- | --- | --- |
| 180-day mortality | OR (95% CI)  P | OR (95% CI)  P | OR (95% CI)  P | OR (95% CI)  P |
| *Crude* | 1.00 (Referent)^a^ | 2.44 (0.74, 8.08)  0.14 | 2.88 (0.90, 9.21)  0.075 | 3.80 (1.22, 11.77)  0.021 |
| *Adjusted^b^* | 1.00 (Referent)^a^ | 2.81 (0.81, 9.74)  0.10 | 3.55 (1.06, 11.84) 0.039 | 4.45 (1.35, 14.69)  0.014 |

Note:

a. Referent in each case is the high functional status group

b. Model 1: Estimates adjusted for age, gender, New York Heart Association Class III/IV,

Chronic Lung Disease, Hypertension, Diabetes, Cerebro-Vascular Disease, and the STS Score
