# Supplementary material for: Decreased preoperative functional status is associated with increased mortality following coronary artery bypass graft surgery
Source: PLoS One. 2018 Dec 13;13(12):e0207883. doi: 10.1371/journal.pone.0207883 (PMC6292581; doi:10.1371/journal.pone.0207883)
Supplement: S4 Appendix — Unadjusted and adjusted associations between functional status groups and 180-day mortality in the parent cohort (N = 6,082). (DOCX) [file pone.0207883.s004.docx]

**S4 Appendix**

**Supplementary Table D.** **Unadjusted and adjusted associations between functional**

**status groups and 180-day mortality in the parent cohort (N= 6,082)**

.

|  |  | **Assessed Functional Status Group** | | |
| --- | --- | --- | --- | --- |
|  | **Not Assessed^a^** | **High** | **Moderate** | **Low** |
| 180-day mortality | OR (95% CI)  P | OR (95% CI)  P | OR (95% CI)  P | OR (95% CI)  P |
| *Crude* | 1.00 (Referent)^b^ | 0.55 (0.24, 1.25)  0.15 | 0.96 (0.60, 1.53) 0.86 | 1.69 (1.01, 2.82)  0.044 |
| *Adjusted^c^* | 1.00 (Referent)^b^ | 0.86 (0.37, 2.00)  0.72 | 1.09 (0.63, 1.90)  0.75 | 1.85 (1.01, 3.40) 0.047 |

Note:

a. Assumption that patients not assessed by physical therapist have independent functional status

a. Referent in each case is the Not Assessed group, assumed to have independent functional status

b. Model 1: Estimates adjusted for age, gender, New York Heart Association Class III/IV,

Chronic Lung Disease, Hypertension, Diabetes, Cerebro-Vascular Disease, and the STS Score.
